# A virus-like particle assembly system for probing the HIV-1 Gag–Pol dimerization domain: supporting evidence for reverse transcriptase involvement in protease activation by influencing Gag–Pol/Gag–Pol interaction

**DOI:** 10.1128/jvi.02236-24

**Published:** 2025-03-12

**Authors:** Shih-Han Hsieh, Kuo-Jung Huang, Chin-Tien Wang

**Affiliations:** 1 Institute of Clinical Medicine, National Yang Ming Chiao Tung University-Yangming Campus 598159 , Taipei, Taiwan; 2 Division of Clinical Research, Department of Medical Research, Taipei Veterans General Hospital 46615 , Taipei, Taiwan; The Ohio State University, Columbus, Ohio, USA

**Keywords:** HIV, retroviruses, reverse transcriptase, Gag-Pol

## Abstract

HIV-1 protease (PR) activation is triggered by Gag–Pol dimerization. We previously reported that reverse transcriptase (RT) amino acid substitution mutations resulted in p66/51RT heterodimer instability associated with impaired PR activation, and that treatment with *efavirenz* (*EFV*, an RT dimerization enhancer) increased PR activation, suggesting RT involvement. However, the contribution of RT to PR activation via the promotion of Gag–Pol dimerization has not been corroborated. To determine whether RT/RT interaction affects Gag–Pol dimerization, RT amino acid substitution mutations known to impair PR activation were cloned into a p6gag-containing construct, Gagp6–Pol, which assembles and releases virus-like particles (VLPs) when PR is inactivated. To map domains involved in Gag–Pol/Gag–Pol interaction, the major Gag assembly domain, with or without additional p6*, PR, or integrase (IN) deletions, was removed from Gagp6–Pol. Resulting constructs were transiently expressed in HEK293T cells. Sucrose density gradient fractionation and electron microscopy results suggest that p6gag-containing RT could form VLPs with lower densities and smaller sizes compared to wild-type particles. RT-PCR results suggest that p6–RT is capable of viral RNA packaging. RT-destabilizing amino acid mutations associated with PR-mediated virus processing deficiencies were found to be capable of reducing Gagp6–Pol VLP yields and attenuating EFV enhancement of Gagp6–Pol VLP assembly. Our results support the proposal that impaired RT stability or RT/RT interaction can disrupt Gag–Pol/Gag–Pol interaction, leading to impaired PR activation. This Gagp6–Pol VLP assembly system offers a potential assay method for probing domains involved in Gag–Pol/Gag–Pol interaction.

**IMPORTANCE:**

HIV-1 protease (PR) activation for mediating virus particle processing is essential for virus infectivity. As part of our attempt to determine whether Gag–Pol dimerization triggers PR activation, we found that RT point mutations that impair RT heterodimer stability and virus particle processing markedly reduced VLP assembly efficiencies in a p6gag-containing Gag–Pol expression vector (designated Gagp6–Pol). Further, these unstable RT point mutations markedly inhibited the facilitating effect of an RT dimerization enhancer on Gagp6–Pol VLP assembly. Our data support the proposal that RT/RT interaction contributes to PR activation by promoting Gag–Pol/Gag–Pol interaction, thus suggesting that targeting Gag–Pol dimerization may serve as an alternative HIV/AIDS treatment strategy. A Gag–Pol VLP assembly assay might be usable for probing the potential impacts of Gag–Pol dimerization on PR activation.

## INTRODUCTION

The retrovirus gag gene is translated as a polyprotein that, by itself, is sufficient for virus-like particle (VLP) assembly and release (1, 2). Three enzymes encoded by the pol gene are required for retrovirus replication as follows: protease (PR), reverse transcriptase (RT), and integrase (IN). In the case of HIV-1, Gag is translated as a Pr55 precursor, whereas Pol is translated as a Pr160gag–pol fusion polyprotein via a −1 ribosomal frameshift from gag to a pol-coding sequence at a frequency of 5%–10% (3). Pr160gag–pol is incorporated into virions via Pr55gag association (4–8). The dimerization or multimerization of Pr160gag–pol triggers embedded PR activation that mediates virus particle maturation by proteolytically processing Pr55gag and Pr160gag–pol. This PR-mediated virus maturation is essential for virus infectivity acquisition (1, 9–12).

Genetic analyses of HIV-1 Gag suggest that most gag-coding sequences are dispensable for VLP assembly and budding. A minimal Gag sequence, containing the p6gag budding domain (13, 14), the C-terminal capsid (CA) domain, including a major homology region (MHR) (15, 16), and an N-terminal myristylation signal sequence (10, 17, 18), is sufficient for efficient VLP assembly (19, 20). Results from our previous studies suggest that Pr160gag–pol viral incorporation is dependent on interaction with Pr55gag via its N-terminal Gag domain (21, 22). Whereas other researchers have shown that HIV-1 Pol is still capable of viral incorporation at a much lower level of efficiency (23, 24), we observed HIV-1 RT packaging into VLPs when expressed with Pr55gag (25). One research team demonstrated the incorporation of human foamy virus Pol into virions without expression as a Gag–Pol fusion protein (26), and another reported the viral incorporation of murine leukemia virus Pol when co-expressed with Gag (27). These data suggest that retroviral Pol or RT is capable of self-association, as well as interaction with Gag.

Within HIV-1 Pr160gag–pol, p6gag is truncated and replaced with a transframe region referred to as p6pol or p6*. We previously described a Pr160gag–pol containing intact p6gag (designated Gagp6–Pol) that is capable of VLP assembly and release (28), thus supporting the hypothesis that the failure of Pr160gag–pol to form VLPs is likely due to an absence of the p6gag budding domain. In turn, mutations impeding Gag–Pol/Gag–Pol interaction may also significantly inhibit Gagp6–Pol VLP assembly efficiency. Given that Gag–Pol dimerization triggers PR activation, the appearance of p66/51RT instability associated with reduced PR-mediated virus processing efficiency (29) suggests that defective RT/RT interaction might impair PR activation by disrupting Gag–Pol dimerization. The purpose of this study is to test the following two hypotheses: that RT mutations associated with defective PR activation decrease Gagp6–Pol VLP assembly by impeding Gag–Pol/Gag–Pol interaction, and that enhanced RT/RT or Gag–Pol/Gag–Pol interaction increases Gagp6–Pol VLP assembly efficiency. We assessed this efficiency in the presence or absence of an RT dimerization enhancer following the insertion of RT mutations known to impair RT stability and PR activation. We also analyzed domains contributing to Gagp6–Pol VLP assembly by combining various Gag–Pol sequence deletions.

## RESULTS

### Mutations impairing RT stability reduce the VLP assembly efficiency of p6gag-containing Gag–Pol molecules

**Fig 1:**
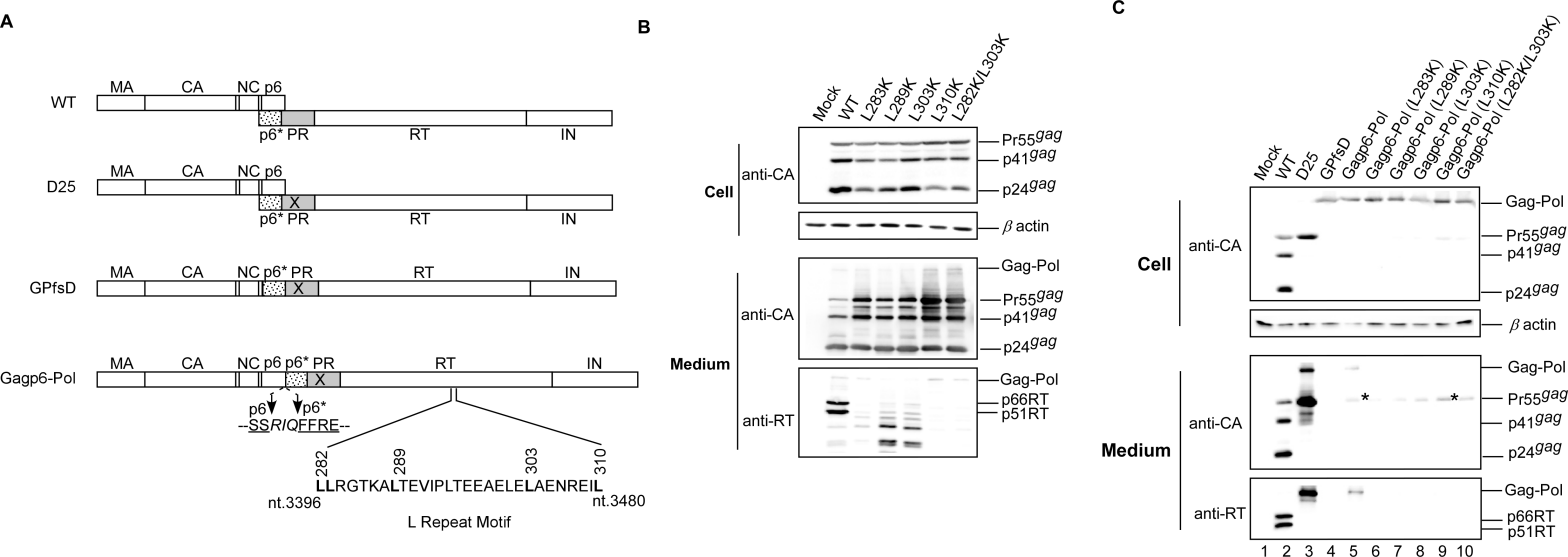
Effects of amino acid substitutions of RT leucine repeat motif residues on RT stability and Gag–Pol virus-like particle assembly. (**A**) Schematic representations of HIV-1 Gag and Gag–Pol expression constructs. Indicated are mature MA, CA, NC, p6, and pol-encoded p6*, PR, RT, and IN. “X” denotes an inactivated PR mutation. RT leucine repeat motif residues are indicated. (**B, C**) Amino acid changes at the RT leucine zipper motif lead to RT instability associated with a defect in virus maturation (panel B) or reduced Gagp6–Pol VLP production (panel C). HEK293T cells were transfected with designated construct plasmid DNA. At 48–72 h post-transfection, cells and supernatants were harvested, prepared, and subjected to western blotting as described in the Materials and Methods section. HIV-1 Gag proteins were probed with an anti-CA (p24gag) monoclonal antibody. Pr160gag–pol, Pr55gag, p41gag, p24gag, and p66/51RT positions are indicated. Asterisks denote a translated Gag precursor variant resulting from incomplete suppression of the gag/pol frameshift signal.

A hydrophobic leucine repeat motif (LRM) spanning RT codons L282 to L310 has been proposed as part of RT/RT interaction (30). We previously found that a lysine replacement of LRM residues led to RT instability associated with reduced PR-mediated virus particle-processing efficiency (29), suggesting that LRM mutations may impair PR activation by disrupting Gag–Pol dimerization. To examine their inhibitory effect on Gagp6–Pol VLP assembly efficiency, the LRM mutations L283K, L289K, L303K, L310K, and a double L282K/L303K mutation previously shown to impair RT stability and virus processing (29) were randomly selected and cloned into Gagp6–Pol (Fig. 1A). The following two constructs served as controls: D25 (a PR-inactivated mutant) and GPfsD (a Gagp6–Pol version lacking p6gag). A wild type (wt), each of the selected LRM mutants, and Gagp6–pol constructs containing the LRM mutations were transiently expressed in HEK293T cells. Supernatants and cells were collected 48–72 h post-transfection, prepared, and subjected to western immunoblot analysis. Similar to our previous report (29), all of the LRM mutants exhibited barely detectable or unstable virus-associated p66/51RT, with reduced efficiency in virus-particle processing compared to the wt (Fig. 1B).

With or without the LRM mutations, GPfsD and Gagp6–Pol both expressed readily detected cellular Gag–Pol precursors. Gag–Pol was detected in Gagp6–Pol transfectant supernatant, but not in GPfsD transfectant samples (Fig. 1C, lane 4 vs lane 5). Gagp6–Pol backbone constructs expressed a Gag precursor variant (Fig. 1C, asterisks), likely due to the incomplete mutation-mediated suppression of the gag–pol frameshift signal. These observations are consistent with previous reports (28). Results from repeat independent experiments indicate that all Gagp6–Pol constructs containing the LRM mutations exhibited barely detectable Gag–Pol in medium (Fig. 1C bottom panel, lanes 6–10). These results support our hypothesis that LRM mutations may impede Gagp6–Pol VLP assembly by disrupting Gag–Pol/Gag–Pol interaction.

### LRM mutations counteract the EFV enhancement effect on Gagp6–Pol VLP assembly

**Fig 2:**
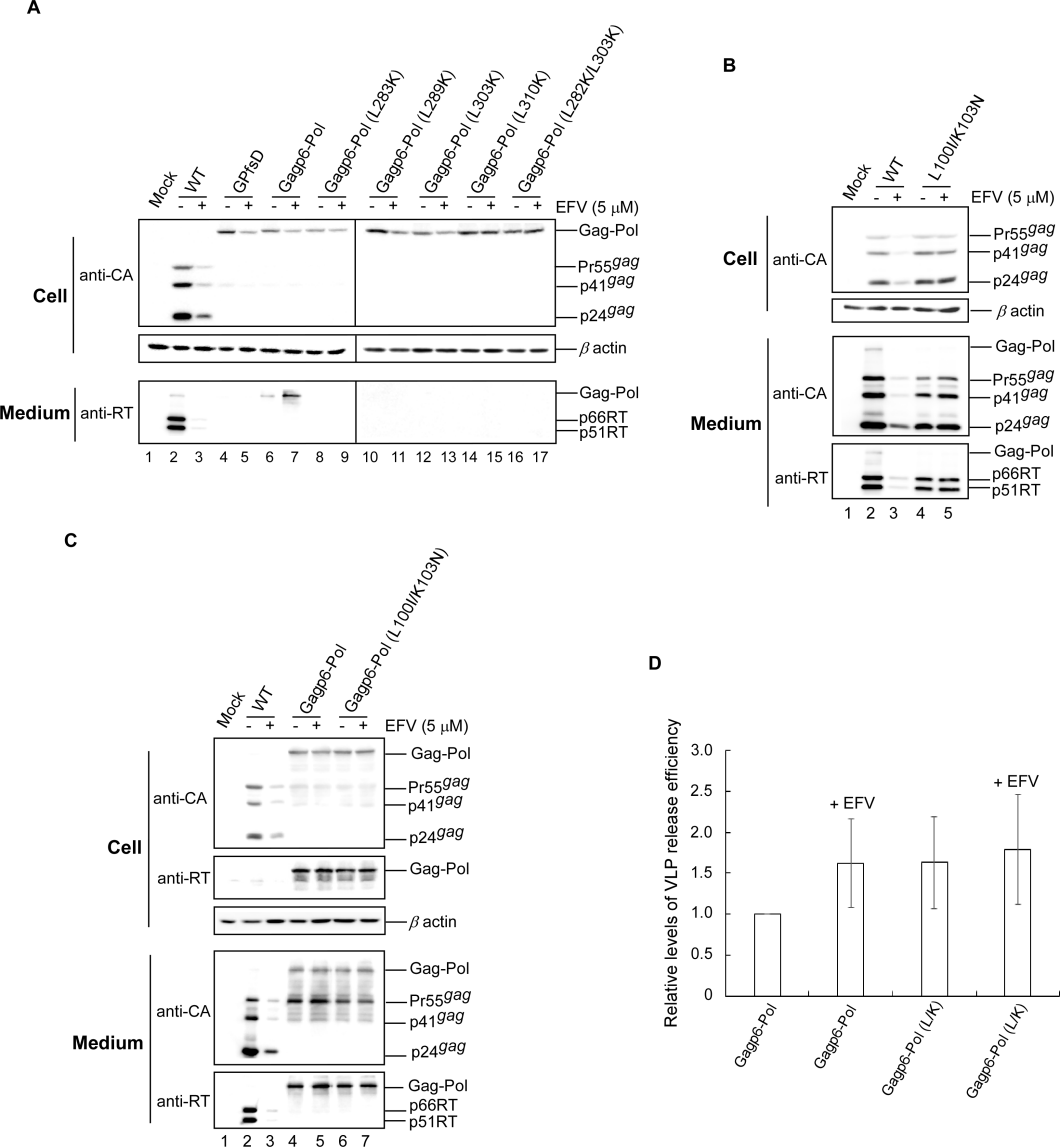
Amino acid changes at RT leucine repeat motif residues counteract EFV enhancement of Gagp6–Pol VLP assembly. HEK293T cells were transfected with wt, GPfsd, Gagp6–Pol, or Gagp6–Pol containing an EFV-resistant mutant (L100I/K103N) or designated amino acid substitution mutation at the RT leucine repeat motif. At 18 h post-transfection, cells were split equally onto two dish plates with or without EFV. At 2–3 days post-transfection, cells and culture supernatants were collected, prepared, and subjected to western immunoblot analyses as described in the Fig. 1 caption. Viral protein levels in both medium and cells for Gagp6–Pol and Gagp6–Pol (L100I/K103N) were quantified by scanning RT-associated band densities from western blots (panel C) in three independent experiments. Medium band densities were divided by medium plus cell band densities for each construct and normalized to those of the Gagp6–Pol group not treated with EFV in parallel experiments (panel D). Bars indicate standard deviation.

Efavirenz (EFV), a non-nucleoside reverse transcriptase inhibitor (NNRTI) with *in vitro* RT dimerization enhancement activity (31–33), reduces virus yields by enhancing PR-mediated Gag cleavage efficiency (33, 34). According to one proposal, EFV may bind to the p66RT domain in a Gag–Pol context, thereby promoting Gag–Pol/Gag–Pol interaction to trigger PR activation (34). We previously reported that LRM mutants counteract the EFV enhancement effect on Gag cleavage (29), likely by diminishing the EFV-associated enhancement of Gag–Pol dimerization. We therefore assumed that EFV is capable of enhancing Gagp6–Pol VLP assembly, with LRM mutations possibly inhibiting the EFV enhancement effect. As expected, EFV treatment triggered an increased Gagp6–Pol VLP yield at the same time that the presence of LRM mutations reduced the EFV enhancement effect on Gagp6-Pol VLP assembly (Fig. 2A lower panel, lanes 6–17). However, quantification results indicate that the EFV enhancement effect on Gagp6–Pol VLP release did not achieve a statistically significant level (Fig. 2C and D). A possible explanation is that the EFV enhancement effect on Gag–Pol dimerization for triggering PR activation is insufficiently strong for significantly promoting the degree of Gag–Pol multimerization required for VLP assembly. Additional control experiments showed that the EFV-resistant mutation L100I/K103N diminished the EFV enhancement effect on Gag processing (Fig. 2B). However, the presence of L100I/K103N did not significantly affect the Gagp6–Pol assembly and release, whether or not EFV treatment was performed (Fig. 2C and D). Combined, the data support our hypothesis that impaired Gag–Pol/Gag–Pol interaction incurred by LRM mutations result in reduced Gagp6–Pol VLP assembly efficiency.

### p6gag domain confers capacity for HIV-1 Pol VLP assembly

**Fig 3:**
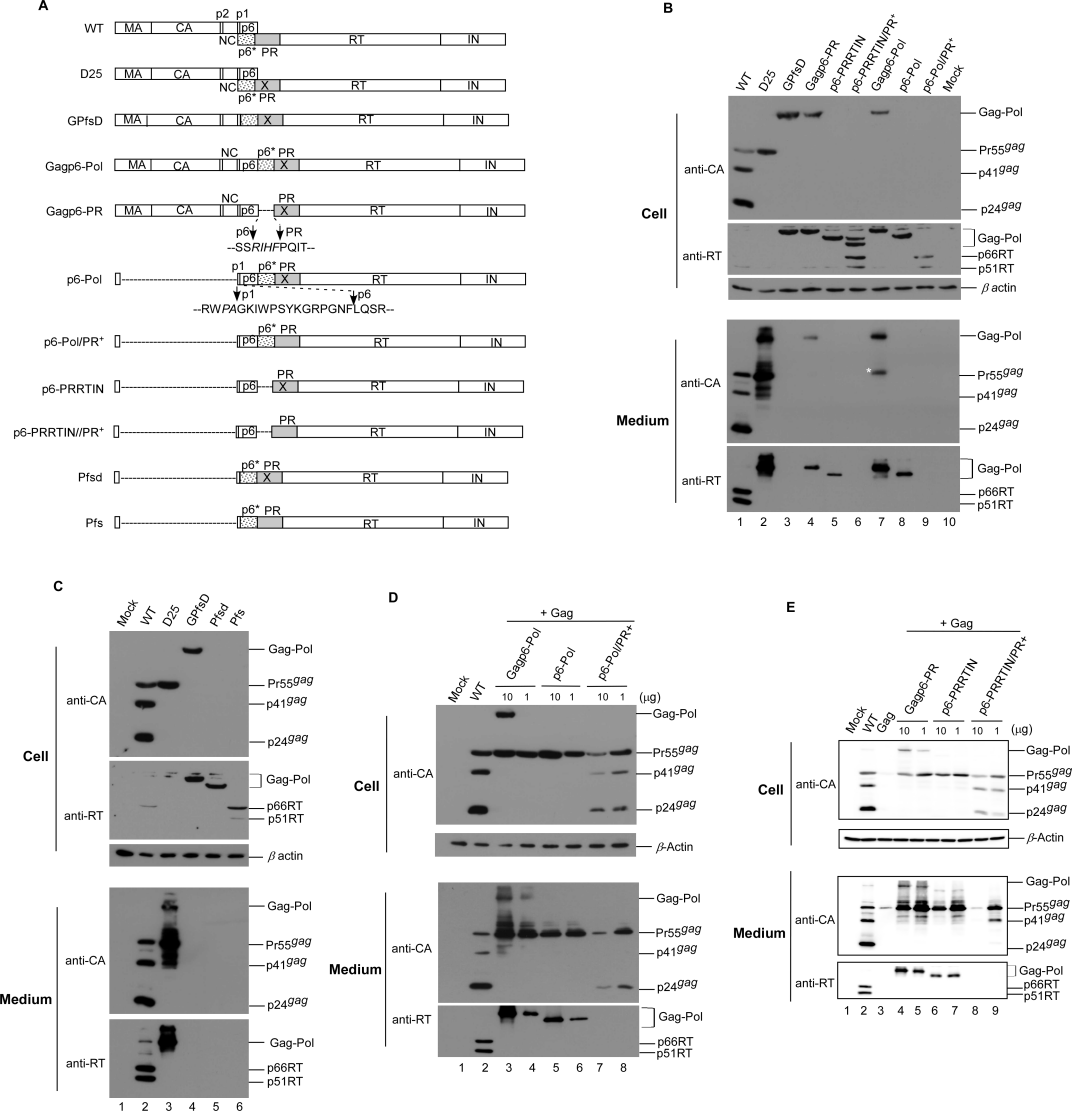
Deletion effects of p6* and/or Gag assembly domains on VLP assembly and PR activity. (**A**) Schematic representations of HIV-1 Gag and Gag–Pol expression constructs. Mature domains from gag and pol-encoded proteins are indicated. Spacer peptides (**P2 AND P1**), respectively, separating NC from CA, and p6 domains are indicated. “X” denotes an inactivated mutation at PR. Dashed lines indicate deleted sequences. Amino acid residues in deleted junctions are shown. Foreign amino acid residues are in italics. (**B, C**) p6* removal from Gagp6–Pol and p6–Pol improve VLP assembly efficiency. HEK293T cells were transfected with the designated plasmid. At 2–3 days post-transfection, supernatants and cells were collected, prepared, and subjected to western immunoblot analyses as described in the Fig. 1 caption. (**D, E**) Incorporation of p6gag-containing Gag–Pol or Pol into Pr55gag particles. HEK293T cells were transfected with 10 µg of Pr55gag expression plasmid alone or combined with 1 or 10 µg of indicated p6gag-containing Gag–Pol or Pol expression plasmids. Plasmid DNA amounts were maintained at 20 µg by the addition of pBlueScript SK. At 48–72 h post-transfection, supernatants and cells were harvested and subjected to western immunoblotting as described above.

Since the Gag domain may be a principal factor in Gagp6–Pol VLP assembly, it is very likely that the removal of Gag from Gagp6–Pol substantially compromises that assembly. To test this idea, we removed most of the gag-coding sequence from Gagp6–Pol while leaving the N-terminal myristylation moiety and p6gag budding domain intact. The resulting construct was designated p6–Pol (Fig. 3A). p6–Pol/PR+ (a PR-active version of p6–Pol) was created to examine the impact of PR activity on VLP assembly. To test the specific effect of transframe region (TFR) p6* on VLP assembly, p6* was removed from Gagp6–Pol, p6–Pol, and p6–Pol/PR+, respectively, yielding the constructs Gagp6–PR, p6–PRRTIN, and p6–PRRTIN/PR+. The p6gag-lacking counterparts of Gagp6pol, p6–Pol, and p6–Pol/PR+ were designated GPfsD, Pfsd, and Pfs, respectively; these served as controls. Each cloned construct was transiently expressed in HEK293T cells. Western immunoblot results indicate substantial amounts of RT-associated products in p6–Pol transfectant supernatant (Fig. 3B bottom panel, lane 8). Combined, the data suggest that the presence of p6gag is still sufficient to promote HIV-1 Pol assembly and release from cells, even without the major Gag assembly domain. As expected, RT-associated products were barely detectable in supernatant samples collected from the non-p6gag group (GPfsd, Pfsd, and Pfs) (Fig. 3C, lanes 4–6). In contrast, p6–Pol/PR+ expressed barely detectable RT-associated products in supernatant, even though cellular p66RT was readily detectable (Fig. 3B, lane 8 vs lane 9). This suggests that inhibited p6–Pol/PR+ assembly and release are likely due to enhanced cleavage associated with overexpressed PR activity.

The removal of p6* from Gagp6–Pol and p6–Pol triggered a noticeable reduction in VLP yields (Fig. 3B, lanes 4–5 vs lanes 7–8), suggesting that the presence of p6* may contribute to Gagp6–Pol and p6–Pol VLP assembly. When co-expressed with Pr55gag, an increase in transfected Gagp6–Pol or Gagp6–PR plasmid DNA resulted in higher virus-associated Gag–Pol levels (Fig. 3D and E), suggesting that increased Gag–Pol levels in the medium are due, at least in part, to Pr55gag particle incorporation via Gag/Gag interaction. An increase in the level of co-transfected p6–Pol plasmid DNA also led to increased p6–Pol levels in the medium, whereas no major changes in p6–PRRTIN levels in the medium were observed following co-transfection with increased amounts of plasmid DNA (Fig. 3D, lanes 5–6 vs Fig. 3E, lanes 6–7). This suggests that the presence of p6* may help p6–Pol incorporate Pr55gag particles. An increase in the amount of PR-native p6–Pol or p6–PRRTIN resulted in a substantial decrease in virus-associated Gag. Note that mature p24gag was readily detected in p6–Pol/PR+ supernatant and cell samples, but not in p6–PRRTIN/PR+ following Pr55gag co-transfection at a DNA ratio of 1:10 (Fig. 3D lane 8 vs Fig. 3E lane 9). This suggests that p6* removal from p6–Pol/PR+ significantly impaired PR activity, which is in agreement with the proposal that p6* plays a modulating role in PR activation (35–41). Combined, the data support the proposal that p6gag confers HIV-1 Pol assembly and release capability. Unless otherwise indicated, all Gag/Gag–Pol expression constructs from this point forward were expressed in a PR-inactivated backbone, since PR activity can markedly affect VLP assembly.

### An intact Gag or p51RT domain is important for Gagp6–Pol VLP assembly

**Fig 4:**
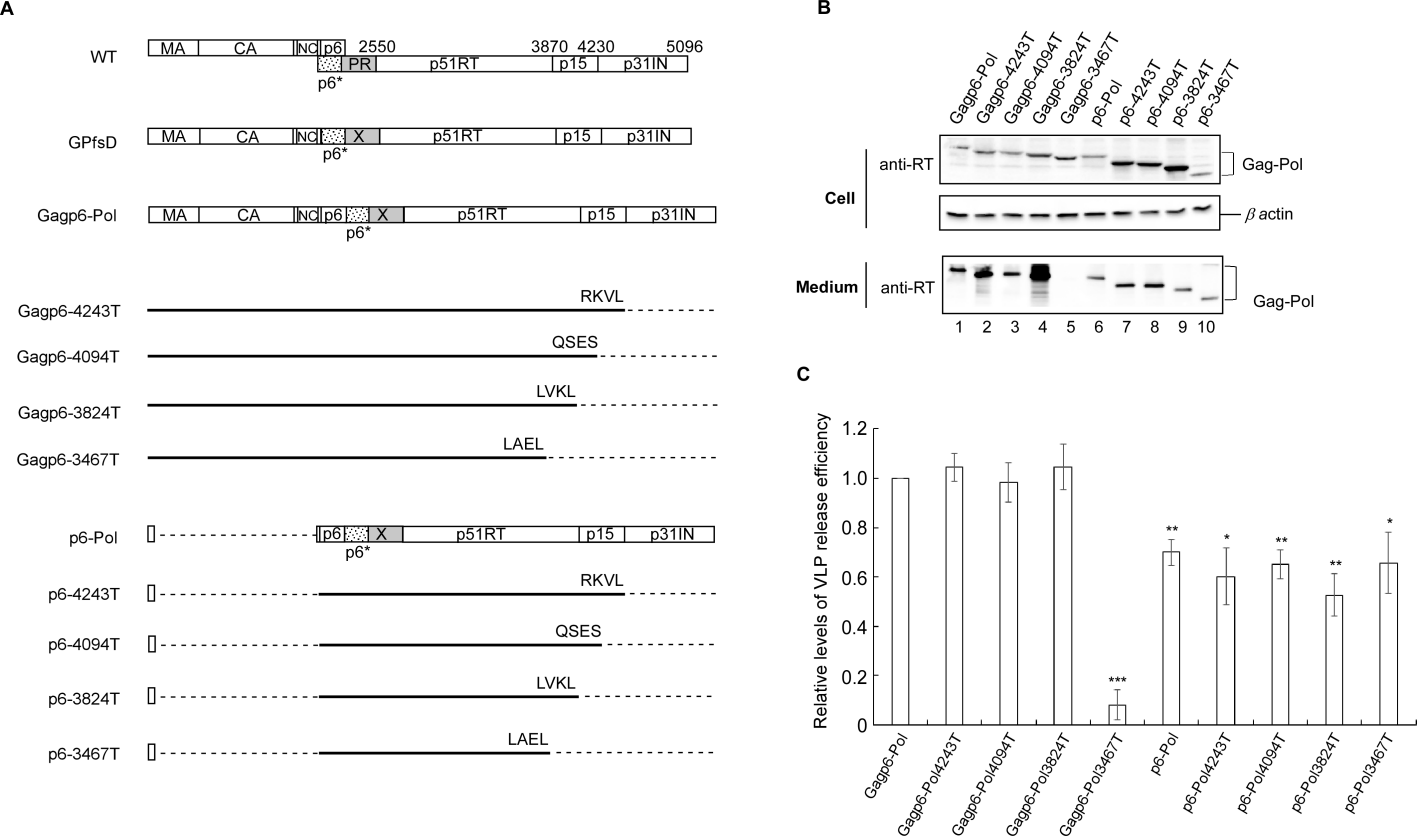
Effects of C-terminal Pol deletions individually or in combination with a Gag assembly domain deletion on VLP assembly. (**A**) Schematic representations of wt or mutant HIV-1 expression constructs. Mature Gag, Pol, and inactivated PR domains are denoted as described above. Numbers in designated constructs denote proviral nucleotide positions with an inserted stop codon. The final four amino acid residues in deleted regions are shown. Dashed lines indicate deleted regions. (**B**) HEK293T cells were transfected with designated constructs. At 48–72 h post-transfection, cells and culture supernatants were collected, prepared, and subjected to western immunoblotting. (**C**) Viral protein levels in both medium and cells were quantified by scanning RT-associated band densities from western blots in three independent experiments. Ratios of medium band densities to medium plus cell band densities were normalized to those of Gagp6–Pol in parallel experiments. Bars indicate standard deviation. *P < 0.05; **P < 0.01; and ***P < 0.001.

We previously reported that C-terminal deletions involving the p51RT domain significantly reduced Gagp6–Pol VLP assembly efficiency (28). Since the results for the present study suggest that p6–Pol is still capable of VLP assembly despite the absence of a major Gag assembly domain, we set out to determine whether additional deletions in the C-terminal Pol sequence also affect the same assembly process. We recombined the Gag–Pol truncation mutations 4243T, 4094T, 3824T, and 3467T (42) with p6–Pol, yielding the constructs p6–4243T, p6–4094T, p6–3824T, and p6-3467T, respectively (Fig. 4A). The Gagp6–4243T, Gagp6–4094T, Gagp6–3824T, and Gagp6–3467T constructs, all of which contain intact Gag domains, served as controls. Each construct was transiently expressed in HEK293T cells. With the exception of Gagp6–3467T, all of the Gag-intact constructs exhibited VLP release efficiency levels comparable to that of Gagp6–Pol (Fig. 4B, lanes 1–5 and Fig. 4C). This finding, which is consistent with previous results (28), suggests that an intact p51RT domain is required for efficient Gagp6–Pol VLP assembly. Note that the Gag-deletion groups also exhibited readily detectable RT-associated products in supernatants (Fig. 4B, lanes 6–10), although at reduced release efficiency compared to Gagp6–Pol (Fig. 4C).

**Fig 5:**
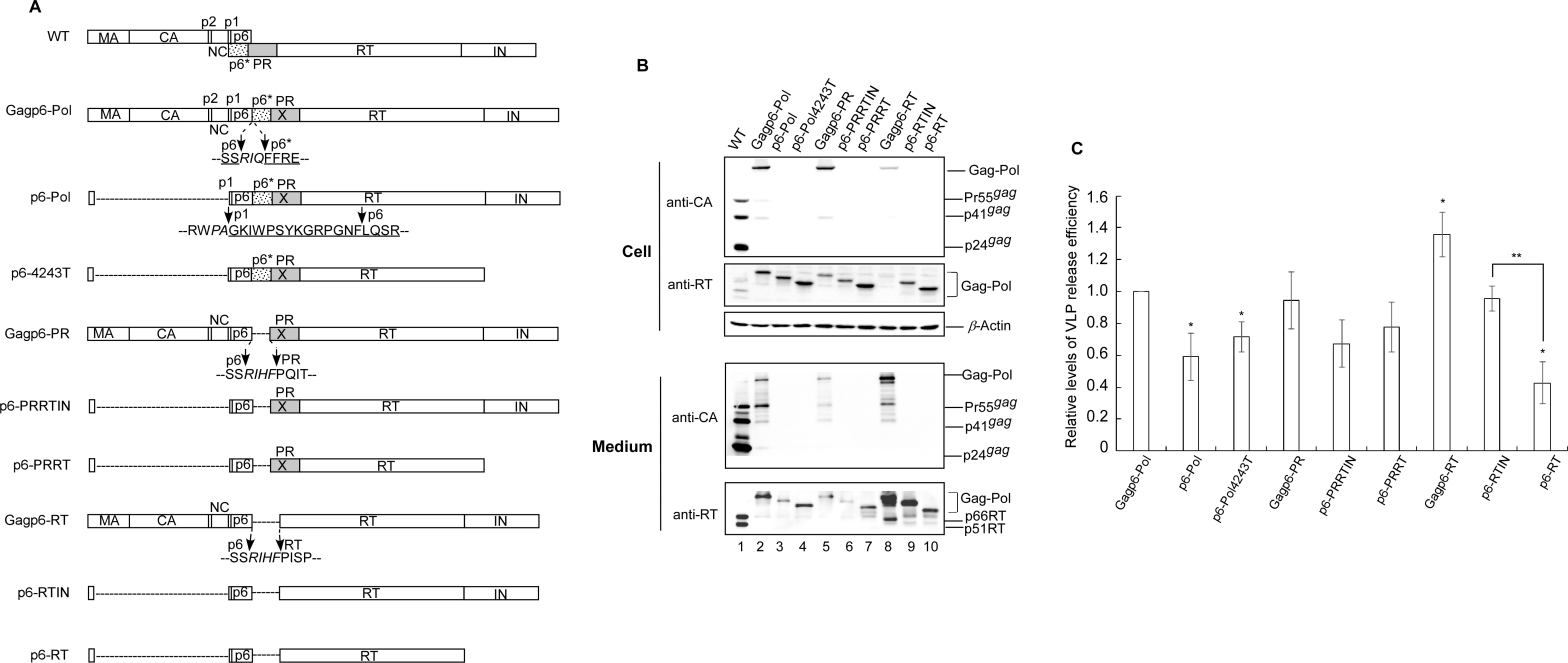
Effects of p6* and/or PR deletions on Gagp6–Pol and p6–Pol VLP assembly. (**A**) Schematic representations of HIV-1 wt and mutant Gag–Pol expression constructs. Mature Gag protein domains and pol-encoded p6*, PR, RT, and IN are indicated. “X” in a PR domain denotes an inactivated mutation. Dashed lines indicate deleted regions. Amino acid residues in deletion junctions are shown. Foreign residues are in italics. (**B**) HEK293T cells were transfected with wt or designated Gag–Pol or Pol expression construct. At 48–72 h post-transfection, culture supernatants and cells were collected, prepared, and subjected to western immunoblot analyses. Gag proteins were probed with an anti-CA monoclonal antibody. RT-associated products were detected with an anti-RT serum. (**C**) Viral proteins in both medium and cells were quantified by scanning RT-associated band densities. The ratios of medium band densities to medium plus cell band densities were determined and normalized to those of Gagp6–Pol in parallel experiments. Bars indicate standard deviation. *P < 0.05; **P < 0.01; and ***P < 0.001.

Since the data in Fig. 3 suggest that removal of the p6* domain results in decreased Gagp6–Pol and p6–Pol VLP yields, we repeated this experiment and examined the impacts of additional PR deletions with or without IN deletions on Gagp6–Pol and p6–Pol VLP assembly. As shown in Fig. 5A, constructs containing a PR deletion from Gagp6–PR and p6–PRRTIN were designated Gagp6–RT and p6–RTIN, respectively. IN removal from p6–PRRTIN and p6–RTIN yielded p6–PRRT and p6–RT. Transient expression results for each construct suggest that p6* removal from Gagp6–Pol did not significantly impact VLP release efficiency (Fig. 5B and C). Note that the Gagp6–RT construct with a PR deletion from Gagp6–PR produced RT-associated products in the medium at a significantly higher level compared to Gagp6–Pol and Gagp6–PR (Fig. 5B lane 8 vs lanes 2 and 5; Fig. 5C). Likewise, p6–PRRTIN VLP yields increased markedly following a PR sequence deletion (Fig. 5B, lane 6 vs lane 9). These data suggest that the presence of a PR sequence might exert a negative impact on Gagp6–Pol and p6–Pol VLP assembly.

### p6-RT VLP assembly

**Fig 6:**
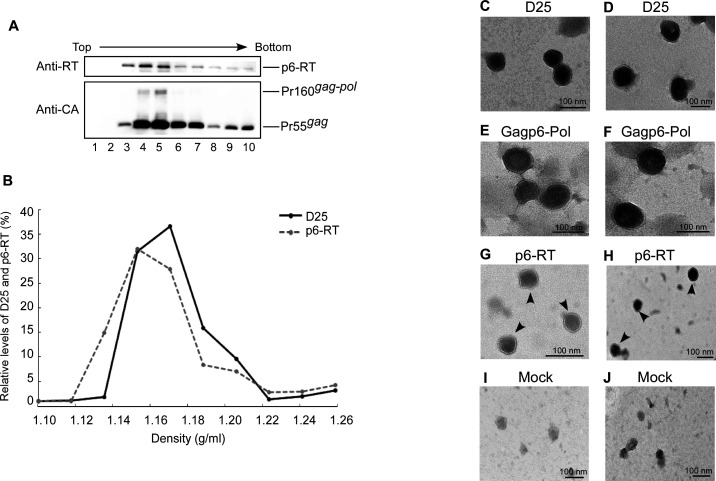
HIV-1 VLP analysis. To make direct comparisons with wt Gag particle densities, resuspended viral pellets containing p6–RT were centrifuged with resuspended viral pellets containing Pr55gag (**D25**) and p6–RT through identical sucrose density gradients (20%–60%, wt/vol). Fractions were collected from top to bottom, measured for sucrose densities, and analyzed with western immunoblots for Pr55gag and p6–RT protein levels (**A, B**). For electron microscope analysis of VLPs, culture supernatants collected at 48 h post-transfection were filtered and pelleted through 20% sucrose cushions. Pellets were resuspended in PBS buffer, stained, and observed with a TEM (**C–J**).

The above results suggest that Gag–Pol constructs containing p6gag, but with large deletions in Gag and Pol, are still capable of self-association and release. Since culture supernatants were centrifuged through 20% sucrose cushions, we believe that viral proteins collected from re-suspended pellets were present as VLPs. Since p6–RT is a minimal p6gag-containing construct capable of VLP assembly and release, we performed sucrose density fraction analyses to confirm the ability of p6–RT to form VLPs and compare p6–RT and wt Gag particle densities. This involved the pooling of re-suspended p6–RT and D25 viral pellets, and centrifuging them through the same sucrose density gradients. Our results indicate a fraction 4 peak of the p6–RT protein with a sucrose density of 1.14 g/mL, and a fraction 5 peak for D25 Pr55gag with a sucrose density of 1.17 g/mL (Fig. 6B). Electron microscopy analyses indicate that D25 and Gagp6–Pol VLPs have electron-dense core particles with approximately 100-nm diameters (Fig. 6C through F). In comparison, p6–RT VLPs displayed smaller electron-dense core particle diameters of approximately 50–70 nm (Fig. 6G and H, arrowheads). These data suggest that p6–RT is capable of VLP formation at lower densities and smaller sizes compared to wt Gag particles.

**Fig 7:**
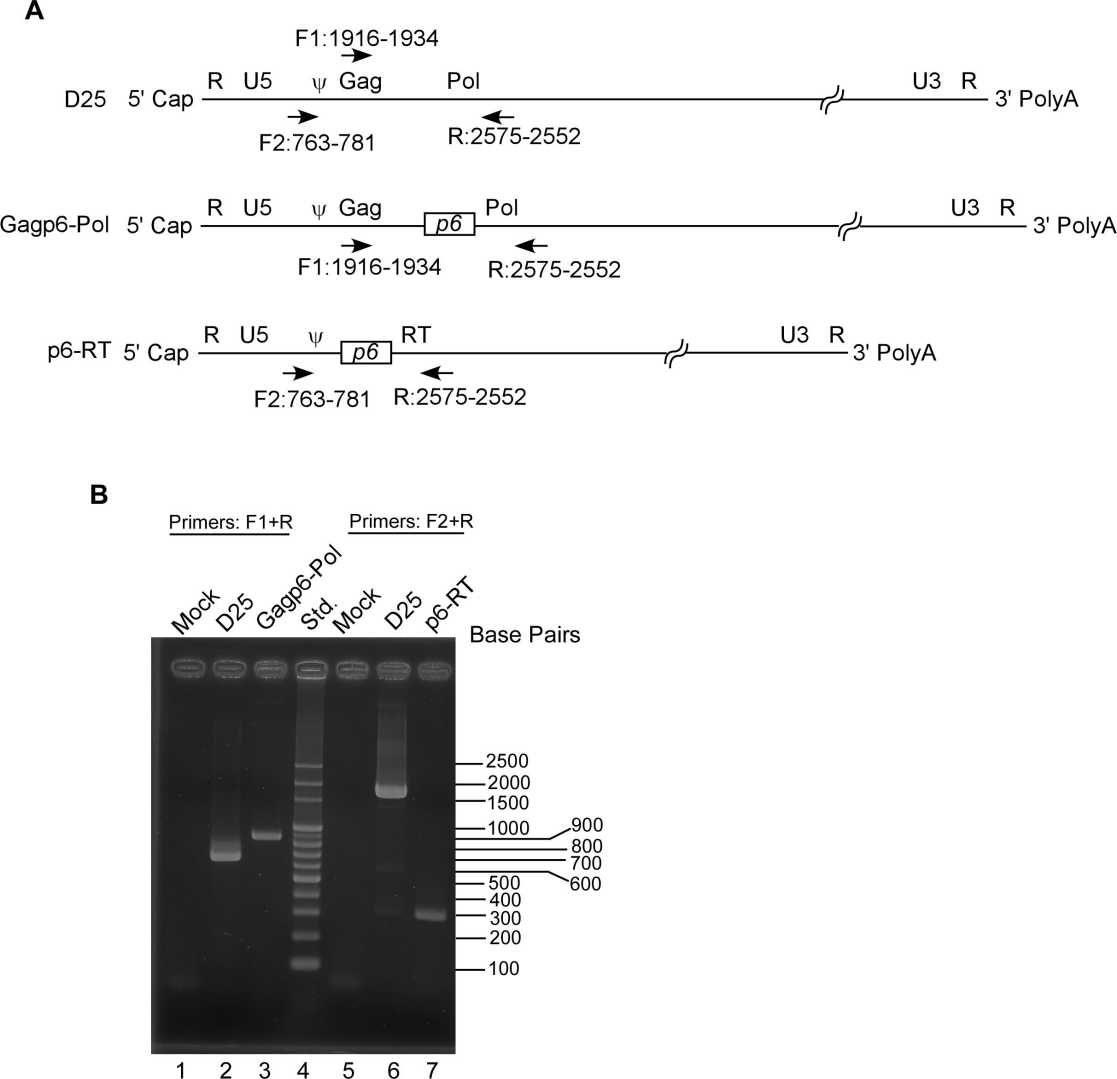
RT-PCR detection of viral RNAs. (**A**) Schematic representations of putative D25, Gagp6–Pol, and p6–RT viral genome structures. Arrows indicate positions of forward and reverse primers used to amplify HIV-1 gene fragments. Ψ denotes the viral genome packaging signal. (**B**) RT-PCR fragment gel electrophoresis. Culture supernatants were collected, filtered, and pelleted through 20% sucrose cushions at 48 h post-transfection. Due to lower release efficiency, p6–RT viral samples were derived from two 10-cm dish plates pooled together. Isolated viral RNAs were subjected to one-step RT-PCR. Amplified fragments were analyzed by agarose gel electrophoresis. Molecular sizes in base pairs are indicated on the right side. Forward and reverse primer sequences: F1, nt 1916-AAT GAT GCA GAG AGG CAA-1934; F2, nt 763-TGA CTA GCG GAG GCT AGA A-781; R, nt 2575-GGT ACA GTC TCA ATA GGG CTA ATG-2552.

We then performed RT-PCR analyses to determine if the lower densities and smaller sizes of p6–RT VLPs are partly due to a defect in viral RNA packaging. To rule out the possibility of plasmid DNA contamination, isolated D25, Gagp6–Pol, and p6-RT viral-associated RNAs were treated with RNase-free DNase prior to repeat RT-PCR experiments. No amplified fragments were found when aliquots of the same isolated RNA samples were directly subjected to PCR in the absence of reverse transcriptase, confirming no plasmid DNA contamination. Due to a large Gag–Pol deletion, the p6–RT-amplified RT-PCR fragments were approximately 1.5 kb shorter than the D25 fragments under the same conditions (Fig. 7, lane 7 vs lane 6). In comparison, the Gagp6–Pol-amplified fragments were approximately 0.2 kb longer than the D25 fragments due to an additional p6gag insertion (lane 3 vs lane 2). These results reduce the likelihood that lower p6-RT particle density is the result of a viral RNA packaging defect.

### Effects of RT amino acid mutations on p6–Pol VLP assembly

**Fig 8:**
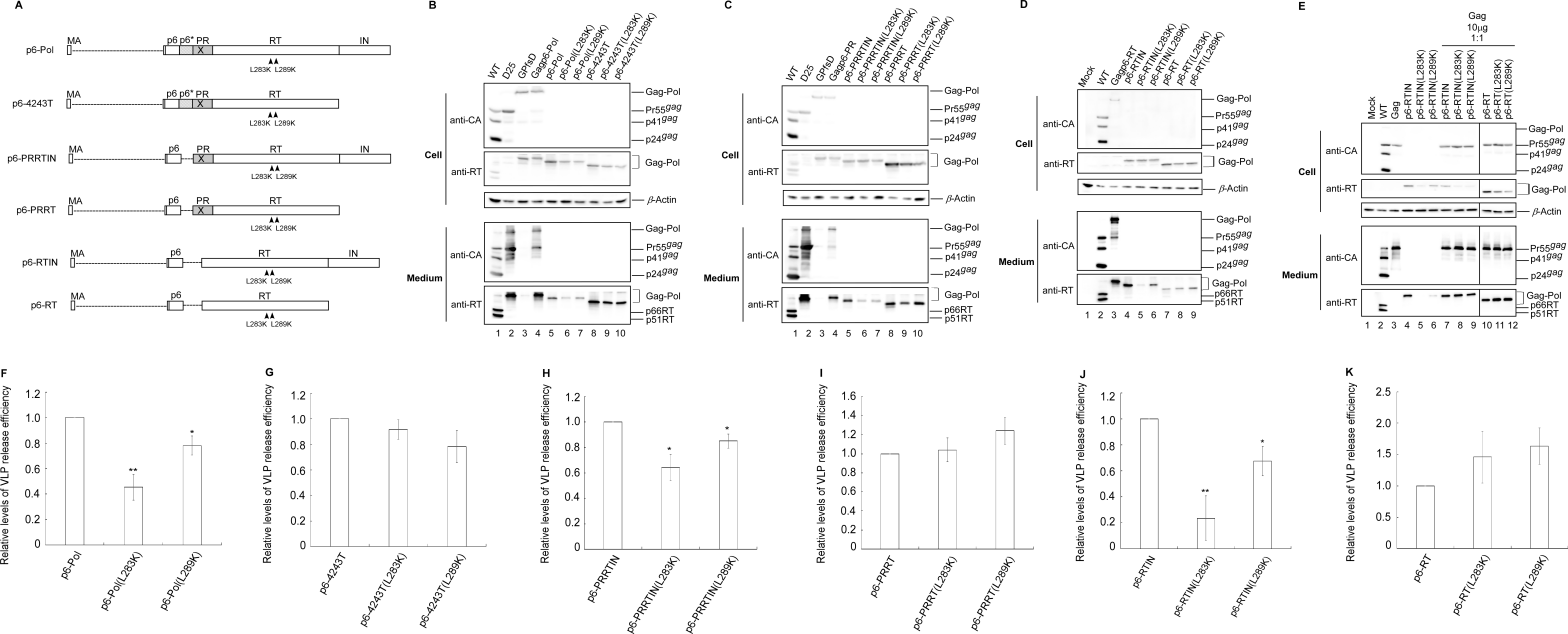
Effects of RT mutations on p6–Pol and p6–RT VLP assembly. (**A**) Schematic representations of p6–Pol constructs with or without various deleted Pol regions as shown in Fig. 5A. Indicated are L283K and L289K RT mutation positions and mature Gag and Pol domains. (**B–E**) HEK293T cells were transfected with a wt or designated mutant construct alone or in combination with equal amounts (10 µg) of the Pr55gag expression plasmid (panel E). At 48–72 h post-transfection, culture supernatants and cells were collected, prepared, and subjected to western immunoblotting. (**F–K**) Viral protein levels in both medium and cells for L283K- and L289K-containing mutants were quantified by scanning RT-associated band densities from western blots (three or four independent experiments) as described in the Fig. 4 legend and normalized to those of their parent constructs in parallel experiments. Bars indicate standard deviations. *P < 0.05; **P < 0.01; and ***P < 0.001.

The results shown in Fig. 1 show that p66/51RT instability, resulting from impaired RT/RT interaction, impeded Gagp6–Pol VLP assembly. Our assumption is that LRM residues may have contributed to Gag–Pol dimerization by stabilizing Gag–Pol/Gag–Pol interaction. Given that the Gag domain is a major Gag–Pol dimerization determinant, LRM mutation effects on Gag–Pol/Gag–Pol interaction can become readily detectable following removal of the Gag domain. To test this hypothesis, the randomly selected mutations L283K and L289K (known to reduce Gagp6–Pol VLP assembly efficiency) were cloned into the Gag-deleted constructs p6–Pol, p6–4243T, p6–PRRTIN, p6–PRRT, p6–RTIN, and p6–RT (Fig. 8A). VLP yields for p6–Pol, p6–PRRTIN, and p6–RTIN were significantly reduced following L283K or L289K insertions (Fig. 8B through D, F, H and J). p6–4243T VLP yields also declined due to the two mutations, but at a statistically non-significant level (Fig. 8B, lanes 8–10; Fig. 8G). In contrast, major impacts were not observed for L283K or L289K on p6-RT assembly in repeat independent experiments (Fig. 8D, lanes 7–9; Fig. 8K). It is possible that IN removal from p6–RTIN exerted a deleterious effect on VLP assembly, but the negative impact of a single amino acid mutation on VLP assembly might be difficult to observe. This would explain, at least in part, why the negative impacts of L283K and L289K on VLP assembly were readily detected for p6–RTIN but not for p6–RT. Results from additional experiments indicate increases in p6–RTIN and p6–RT levels in supernatants when co-expressed with Pr55gag, regardless of whether or not they contained the L283K or L289K mutation (Fig. 8E). Combined, these data suggest that p6–RT and p6–RTIN are capable of efficiently associating with Pr55gag despite the absence of a major Gag assembly domain and that the presence of L283K or L289K does not exert major impacts on RT–Pr55gag association.

## DISCUSSION

Adequate Gag–Pol/Gag–Pol interaction is essential for protease (PR) activation, but Gag–Pol multimerization may still be possible despite the presence of mutations. Our data indicate that mutations responsible for impaired PR-mediated virus particle processing also significantly decrease the efficiency of Gagp6–Pol VLP assembly. In other words, mutations that disrupt Gag–Pol/Gag–Pol interaction leading to PR activation are sufficient for blocking Gagp6–Pol VLP assembly, thus making assembly efficiency a potential marker of the status of Gag–Pol/Gag–Pol interaction.

Our observation that the RT dimerization enhancer and antiretroviral medication EFV substantially enhances Gagp6–Pol VLP assembly (Fig. 2) supports the hypothesis that EFV binding with Gag–Pol results in enhanced Gag–Pol/Gag–Pol interaction (34). Further, RT mutations that impair p66/51RT stability may reduce Gagp6–Pol VLP assembly efficiency and the associated EFV enhancement effect (Fig. 1 and 2). These data suggest that RT LRM mutations block Gag–Pol/Gag–Pol interaction, thus supporting the hypothesis that either RT stability or an RT/RT interaction defect might induce defective Gag–Pol dimerization, leading to impaired PR activation (29).

As we previously reported, C-terminal Pol truncation mutants lacking an intact p51RT domain (3467T) are severely defective in PR-mediated Gag cleavage; in comparison, Gag cleavage efficiency is not significantly affected by the Pol truncation mutants 4243T, 4094T, and 3824T, all of which contain intact p51RT domains (42). This suggests that the Pol truncation mutation 3467T may significantly impair Gag–Pol dimerization, resulting in insufficient PR activation. In the present study, we found that Gagp6–3467T with a C-terminal deletion involving p51RT (corresponding to the 3467T mutant) produced barely detectable VLPs. In contrast, 4243T, 4094T, and 3824T (none of which exerted negative impacts on Gag cleavage) (42) did not exhibit any negative effects on VLP assembly when cloned into the Gagp6–Pol construct (Fig. 4). These results support the idea that Gag–Pol dimerization associated with PR activation modulation can be inferred from the VLP assembly efficiency of Gagp6–Pol.

Significant reductions in Gagp6–Pol VLP assembly efficiency occurred following p6* removal (Fig. 3), suggesting that p6* contributes to Gag–Pol/Gag–Pol interaction, and supporting the idea that p6* plays a role in PR activation regulation, likely through an effect on Gag–Pol dimerization. Further, p6* removal inhibited the power of p6–Pol/PR+ to mediate Pr55gag particle processing and p6–Pol VLP assembly efficiency (Fig. 3), suggesting p6* involvement in Pol/Pol interaction associated with PR activation and p6–Pol VLP assembly.

RT that contains p6gag (p6–RT) is still capable of VLP self-association and release, although at lower densities and smaller sizes compared to wt virus particles (Fig. 6). The qualitative RT-PCR detection of p6–RT VLP-associated RNAs suggests that p6–RT is still capable of packaging viral RNA in the absence of a nucleocapsid (NC) domain (Fig. 7). The involvement of p6gag in viral genome packaging has not been proven (43). Some studies suggest that p6gag is required for viral genome association (44, 45); others argue that the viral genome packaging process is p6gag independent (46). The possibility also remains that the RT domain contributes to viral RNA packaging in a p6–RT context.

If the p66/51RT instability observed in LRM mutations was due to unstable RT/RT interactions as incurred by those mutations (Fig. 1), it is possible that the LRM mutations reduced p6–RT VLP assembly efficiency by disrupting RT self-association. However, two randomly selected LRM mutations (L283K and L289K) did not appear to exert negative impacts on the p6–RT VLP assembly or association with Pr55gag (Fig. 8D, E and K). It is possible that L283K and L289K did not trigger sufficient perturbations to affect RT self-association in a p6–RT context, but are capable of destabilizing or disrupting the p66/51RT heterodimer that renders RT to viral protease degradation. It is unknown whether the other LRM mutations exerted any negative impacts on p6–RT VLP assembly. However, the negative effects of L283K and L289K on VLP assembly were observed in the contexts of Gagp6–Pol (Fig. 1 and 2), p6–Pol (Fig. 8B and F), p6–PRRTIN (Fig. 8C and H), and p6–RTIN (Fig. 8D and J), all containing an intact IN domain. Apparently, the presence of IN allowed the LRM mutations to trigger conformations that blocked Gagp6–Pol and p6–Pol VLP assembly.

## MATERIALS AND METHODS

### Plasmid construction

The p6gag-containing Gag–Pol expression constructs Gagp6–Pol, Gagp6–PR, and Gagp6–RT are as previously described (28). PCR-mediated overlap extension was used to remove a Gag-coding sequence (proviral nt.831-ClaI to nt.2096-BglII) from Gagp6–Pol, yielding a p6–Pol construct. p6–Pol was then recombined with Gagp6–PR and Gagp6–RT to generate p6–PRRTIN and p6–RTIN, respectively. Previously constructed Pol-truncated clones 4243T, 4094T, 3824T, and 3467T (29, 42) were recombined with p6–Pol, p6–PRRTIN, or p6RT to yield p6–4243T, p6–4094T, p6–3824T, p6–3467T, p6–PRRT, and p6–RT. All engineered constructs were confirmed by DNA sequencing or restriction enzyme digestion. An env-deleted and replication-defective HIV-1 Gag/Gag–Pol expression vector served as a backbone.

### Cell culture and transfection

HEK293T cells were maintained in Dulbecco’s modified Eagle medium (DMEM) supplemented with 10% fetal calf serum. Confluent 293T cells were trypsinized and split at a ratio of 1:10 onto 10-cm dish plates 18–24 h prior to transfection. For each construct, 293T cells were transfected with 20 µg of plasmid DNA using a calcium phosphate precipitation method. Chloroquine (50 µm) was added to culture medium to enhance transfection efficiency.

### Western blotting

Western blot analyses of viral Gag/Gag–Pol expression followed previously described protocols (28). Briefly, cell culture supernatant was filtered through 0.45-µm pore size filters 48–72 h post-transfection, followed by centrifugation through 2 mL of 20% sucrose in TSE (10 mM Tris/HCl, pH 7.5, 100 mM NaCl, 1 mM EDTA) containing 0.1 mM PMSF at 4°C for 40 min at 274,000 × *g* (SW41 rotor at 40,000 rpm). Viral pellets were suspended in lysis buffer IPB (20 mM Tris HCl pH 7.5,150 mM NaCl, 1 mM EDTA, 0.1% SDS, 0.5% sodium deoxycholate, 1% triton-X 100, 0.02% sodium azide) plus 0.1 mM PMSF. Cells were rinsed with ice-cold phosphate-buffered saline (PBS), collected in 1 mL of PBS, pelleted, and resuspended in 250 µL of IPB plus 0.1 mM PMSF prior to microcentrifugation at 4°C for 15 min at 13,700 × *g* to remove cell debris. Supernatant or cell samples were mixed with equal volumes of 2× sample buffer plus 5% β-mercaptoethanol and boiled for 5 min. Samples were subjected to 10% SDS-PAGE and electroblotted onto nitrocellulose membranes. HIV-1 Gag-associated proteins were probed with a primary anti-CA (p24gag) monoclonal antibody (mouse hybridoma clone 183-H12-5C). Membrane-bound RT-associated proteins were detected by rabbit antisera (ab63911) or mouse anti-RT monoclonal antibody (47, 48). A sheep anti-mouse or goat anti-rabbit horseradish peroxidase-conjugated antibody served as a secondary antibody (Jackson ImmunoResearch). Membrane-bound proteins were detected using an enhanced chemiluminescence (ECL) detection system (SuperSignal West Pico chemiluminescent substrate; Thermo Fisher Scientific). Chemiluminescent signals were captured on film or with a UVP Chemstudio Plus Digital Imaging System (Analytik Jena).

### Sucrose density gradient ultracentrifugation

At 48–72 h post-transfection, cell culture supernatant was filtered and centrifuged through 2 mL of 20% sucrose as described above. Viral pellets were resuspended in TSE buffer, loaded to the top layer of a 20%–60% sucrose density gradient, and centrifuged at 274,000 × *g* for 16 h at 4°C (SW50.1 rotor at 40,000 rpm). Fractions (500 µL) were collected from top to bottom for sucrose density measurements. Proteins in each fraction were precipitated with 10% TCA at 4°C for 15 min. Precipitates were rinsed with PBS, resuspended in sample buffer, and subjected to western immunoblot analysis.

### Electron microscopy

Following centrifugation through 20% sucrose cushions, viral samples were loaded onto carbon-coated and UV-treated 200 mesh copper grids for 2 min as previously described (28). Specimen grids were rinsed in sterile filtered water for 15 secs, placed on filter paper to dry, and stained with 1.3% uranyl acetate for 1 min. Excess liquid was drained with filter paper at the edge of each grid, which was thoroughly dried prior to transmission electron microscopy with a JEOL JEM-2000 EXII. EM images were captured at 120,000×.

### RNA extraction and RT-PCR

Virus-associated RNAs were isolated using a spin column-based QIAamp Viral RNA extraction kit according to the manufacturer’s protocols (Qiagen). Isolated viral RNAs were treated with RNase-free DNase followed by one-step RT-PCR using an Invitrogen Superscript III one-step PCR kit. Cycling conditions were the same as described by Gupta et al. (49).

## Data Availability

All data analyzed or generated in this study are presented here and are available on request.
